# Enteric Virome Sensing—Its Role in Intestinal Homeostasis and Immunity

**DOI:** 10.3390/v10040146

**Published:** 2018-03-23

**Authors:** Rebecca N. Metzger, Anne B. Krug, Katharina Eisenächer

**Affiliations:** Institute for Immunology, Biomedical Center, Ludwig-Maximilians-University Munich, Großhaderner Str. 9, D-82152 Martinsried, Germany; rebecca.metzger@med.lmu.de (R.N.M.); anne.krug@med.lmu.de (A.B.K.)

**Keywords:** dendritic cells, macrophages, mononuclear phagocytes, intestinal epithelial cells, type I interferon, intestine, Toll-like receptors, RIG-I-like receptors, microbiome, enteric innate immunity

## Abstract

Pattern recognition receptors (PRRs) sensing commensal microorganisms in the intestine induce tightly controlled tonic signaling in the intestinal mucosa, which is required to maintain intestinal barrier integrity and immune homeostasis. At the same time, PRR signaling pathways rapidly trigger the innate immune defense against invasive pathogens in the intestine. Intestinal epithelial cells and mononuclear phagocytes in the intestine and the gut-associated lymphoid tissues are critically involved in sensing components of the microbiome and regulating immune responses in the intestine to sustain immune tolerance against harmless antigens and to prevent inflammation. These processes have been mostly investigated in the context of the bacterial components of the microbiome so far. The impact of viruses residing in the intestine and the virus sensors, which are activated by these enteric viruses, on intestinal homeostasis and inflammation is just beginning to be unraveled. In this review, we will summarize recent findings indicating an important role of the enteric virome for intestinal homeostasis as well as pathology when the immune system fails to control the enteric virome. We will provide an overview of the virus sensors and signaling pathways, operative in the intestine and the mononuclear phagocyte subsets, which can sense viruses and shape the intestinal immune response. We will discuss how these might interact with resident enteric viruses directly or in context with the bacterial microbiome to affect intestinal homeostasis.

## 1. The Enteric Virome in Health and Disease

The microbiome comprises all microorganisms inhabiting the human body and is a substantial part of our physiology. The term microbiome was defined by Joshua Lederberg as “the ecological community of commensal, symbiotic, and pathogenic microorganisms that literally share our body space” [1]. Over the past decade, the availability of new technologies to study complex microbial communities revealed that the microbiome imparts phenotypic differences between individuals similar to gene variants in the host genome [2]. In particular, the commensal bacteria were attributed to modulate our biology since every human body is colonized on mucosal surfaces and skin by an immense number of diverse strains, which differ between individuals and interact with the host but also with each other over a long period of time [3]. Many studies have examined the composition and the function of the microbiome in our intestine and other body sites including the enteric virome [4]. This review will bring commensalism of enteric viruses into the context of virus sensing and immune regulation in the intestine and its consequences for health and disease.

Intensive characterization over the past decades has revealed that the intestinal microbiome confers beneficial functions to the host by aiding in digestion of food products, maintaining intestinal barrier function, protecting from pathogenic microbial infections and shaping the immune system (reviewed in [5]). However, it became evident that the microbial community in the intestinal lumen, which is regulated by environmental, dietary and host genetic factors not only contains more than 1000 different bacterial species but also a wide variety of viruses, fungi and archaea. The enteric virome encompasses all nucleic acids (DNA and/or RNA) obtained from fecal samples or virus-like particles enriched from fecal samples, which can be mapped to viral genomes [6,7]. We will use the term enteric virome here to encompass all viruses and bacteriophages residing in the intestine. However, a large proportion of the sequences obtained from virus-like particle preparations does not map to any sequences within the public databases indicating that the enteric virome contains many uncharacterized viruses [6]. The enteric virome of humans contains viral genes associated with viruses that infect eukaryotic cells, viruses that infect bacteria (bacteriophages) and even viruses that infect plants derived from the diet of the host [8]. In a recent study by Norman et al. 2.7% of all mapped reads obtained by metagenomic sequencing of human stool samples were viral sequences compared to 50.9% bacterial, 7.2% eukaryotic and 37.2% unassigned sequences. 15% of the viral sequences could be assigned to viral taxonomy—the majority to bacteriophages of the *Caudovirales* and *Microviridae* taxa [9].

Recent studies have shed light on how resident enteric viruses may affect host physiology beyond causing disease [7,10,11,12]. An interesting question is if enteric viruses, which have been detected in metagenomic analyses of fecal samples can be truely viewed as “commensal” gut-resident viruses. For bacteriophages this seems to be clear as they infect bacteria which themselves form stable communities in the intestine. But eukaryotic viruses can only replicate inside of host cells and trigger immune responses, which can inhibit their replication and may or may not clear the infection. Therefore, eukaryotic enteric viruses, whose nucleic acid sequences are repeatedly detected by metagenomic analyses in the feces of healthy humans over time can be derived from acute recurrent infections, chronic persistent infections or reactivation of latent viruses [8]. Longitudinal studies of intestinal viromes in human healthy adult monozygotic twins and their mothers indicate that individual viromes are unique, quite stable and dominated by temperate phages. Despite low intra-individual variability, the enteric virome is affected by developmental changes in early life, which are influenced by environmental factors such as nutrition [13,14].

### 1.1. Eukaryotic Enteric Viruses

Although eukaryotic viruses are rare within the enteric virome of healthy adults, they could also be detected in the aforementioned metagenomic studies and earlier studies [15,16] and comprise single-stranded (ss) RNA, ssDNA, double-stranded (ds) DNA viruses and retroviruses. Constant shedding of enteric eukaryotic viruses in healthy infants was confirmed by PCR for adenoviruses, anelloviruses, bocaviruses, enteroviruses, parechoviruses and picobirnaviruses [17]. Sequences from the eukaryotic virus genera *Circoviridae*, *Anelloviridae*, *Picobirnaviridae*, *Picornaviridae* (including entero-, kobu- and parechoviruses), *Parvoviridae* (mainly bocaviruses), and also *Reoviridae* (rotavirus) were frequently detected in virus-enriched preparations from a control group of 11 healthy children in a recent longitudinal study [18], demonstrating that also viruses which are considered pathogenic frequently reside in the human intestine without causing symptomatic disease. It was also found that asymptomatic people can shed norovirus for longer time periods [19] and specific murine norovirus (MNV) strains were found to persist in the intestine of mice lifelong without causing disease [20]. Thus, even eukaryotic viruses, which are considered pathogens or opportunistic pathogens, are frequently part of the enteric virome of healthy humans and participate in shaping intestinal physiology. Therefore, it is clear that eukaryotic viruses resident in the intestine must be tightly controlled by local defense mechanisms and by the innate and adaptive immune system to prevent development of intestinal pathology.

Resident enteric viruses maintaining low level immune stimulation in the intestinal mucosa have important protective and immunoregulatory effects on the intestine as shown recently in mice persistently infected with MNV [11]. During persistent infection, for example, with MNV strain CR6, small numbers of intestinal epithelial cells (IECs) are a reservoir for MNV and shed the virus [21]. The persistence of MNV in IECs requires the non-structural protein NS1 from strain CR6, which interferes with the antiviral control exerted by type III interferon (IFN) [21,22]. It was shown recently that infection with MNV CR6 can reverse the intestinal abnormalities observed in germ-free and antibiotic-treated mice, thus acting in a manner similar to commensal bacteria [11]. MNV infection increased the size of villi and crypts in the small intestine, restored Paneth cell function, increased the number and function of lymphocytes in the lamina propria and mesenteric lymph nodes (mLNs) (IFN-γ and IgA production) and prevented the expansion of innate lymphoid cells (ILC) type 2 while increasing the number of interleukin (IL)-22 producing ILC type 3. These effects were largely dependent on type I IFN signaling but could not be fully mimicked by systemic application of polyI:C, a potent inducer of type I IFNs [11]. The nonredundant role of enteric viruses for intestinal homeostasis was also demonstrated recently by treating mice orally with a cocktail of antiviral drugs (ribavirin, lamivudine, acyclovir), which inhibit the replication of DNA and RNA viruses as well as retroviruses. Mice pretreated with these antivirals suffered from more severe colitis after exposure to the chemical dextran sodium sulfate (DSS), which severely damages the intestinal epithelium and induces acute inflammation in the colon [12]. Interestingly, the oral antiviral treatment led to a modest reduction in enteric viral load along with an increase in bacteriophages of the *Caudovirales* family [12], resembling the pattern of changes in the enteric virome observed in Crohn’s disease and ulcerative colitis patients [9].

The beneficial and detrimental effects of resident enteric viruses are however very close together and immunodeficiency or host genetic factors affecting epithelial barrier function or immune regulation in the intestine can tip the balance towards pathologic effects of viruses residing in the intestine [23,24,25,26]. For example, mice with a hypomorphic mutation in the autophagy gene *Atg16L1*, which is a susceptibility gene in Crohn’s disease, show Paneth cell abnormalities leading to defective antibacterial defense and a hyperinflammatory state in the intestine [23]. This phenotype is only seen in conventionally housed mice, which are persistently infected with MNV or in mice, which were inoculated with the persistent MNV CR6 strain. Upon exposure to DSS in the drinking water, MNV-infected *Atg16L1*-mutant mice developed increased intestinal pathology in the colon and blunting of villi in the ileum resembling Crohn’s disease [23]. The observed aberrant intestinal pathology was mediated by tumor necrosis factor (TNF)-α and IFN-γ, and required commensal bacteria. Interestingly, IL-10-deficient mice rapidly developed colitis weeks after infection with MNV, which was associated with impaired epithelial barrier function and required the enteric microbiome [27]. Thus, although resident enteric viruses such as MNV clearly provide protection against injury, inflammation and bacterial infection in the intestine [11], they can also impair intestinal barrier function and promote inflammation in genetically susceptible hosts with defects in local defense mechanisms or immune regulation [23,27].

Investigation of the enteric virome in human immunodeficiency virus (HIV)-infected patients or simian immunodeficiency virus (SIV)-infected macaques with acquired immune deficiency syndrome (AIDS) revealed an expansion of the enteric virome and outgrowth of several potentially pathogenic eukaryotic enteric viruses including *Picornaviridae*, *Parvoviridae*, *Anelloviridae* and *Adenoviridae* while bacteriophages were less affected [24,25,26]. Especially the expansion of enteric adenovirus sequences, which was paralleled by an expansion of *Enterobacteriaceae* was linked to AIDS-associated enteropathy and rapid disease progression [24,25,26]. An expansion of persistent DNA viruses including anelloviruses, herpesviruses, papillomaviruses and polyomaviruses as well as dsRNA picobirnaviruses inversely correlated with a decrease in phage richness in the enteric virome of immunocompromised patients within the first 6 weeks after hematopoietic stem cell transplantation (HSCT) [28]. Interestingly, the detection of picobirnaviruses was predictive of graft-versus-host disease (GvHD) suggesting that persistent infection with these viruses may promote GvHD [28]. Taken together, these results demonstrate the pathogenic potential of the enteric virome when it is not sufficiently controlled by the immune system.

### 1.2. Bacteriophages—Potential Role for Modulating Intestinal Physiology and Immunity

Despite the incomplete picture of the taxonomic structure of the mammalian virome, it is clear that the majority of viruses residing in the intestine are not eukaryotic viruses but bacteriophages, including prophages stably integrated into the bacterial genome and lytic phages, which infect and lyse bacteria and can be released from them in response to stress signals [8]. By shaping the composition and function of the bacterial microbiome, bacteriophages indirectly affect intestinal physiology and the development and function of the intestinal immune system [29,30]. Indeed Norman and colleagues found that increased bacteriophage taxonomic richness with an expansion of *Caudovirales* bacteriophages inversely correlated with decreased bacterial richness and diversity (dysbiosis) in inflammatory bowel disease (IBD) patients compared to controls living in the same households [9]. Barr et al. showed that phages adhere to the mucus by interacting with glycan residues within mucin glycoproteins and thus are enriched at mucosal surfaces. Together with antimicrobial peptides in the mucus layer they reduce the density of commensal bacteria in the mucus and provide protection against bacterial pathogens thereby establishing a symbiotic relationship with the host [31].

In addition to their effects on the bacterial microbiome and support of the epithelial barrier function, it is possible that bacterial cell wall components and bacterial and phage DNA, which are released as a consequence of the lysis of bacteria by phages, trigger pattern recognition receptors (PRRs) in epithelial cells or immune cells with contact to the lumen thereby influencing intestinal homeostasis and immunity [8]. Alternatively, bacteriophages could directly trigger responses in eukaryotic cells including epithelial cells and immune cells in the intestine. It was shown that bacteriophages transcytose through epithelial cell layers in vitro [32]. They can also translocate through the intestinal epithelium when applied orally to humans or mice for therapeutic purposes [8,33] and can trigger weak humoral immune responses [34]. Indeed, bacteriophages are found in sterile tissue sites of healthy humans [35]. There is evidence from in vitro studies that phages can attach to the membrane of leucocytes, are internalized by phagocytosis or endocytosis and are degraded in lysosomes, but do not induce inflammatory cytokine production [36]. However, therapeutic targeting of phages to B16 melanomas in mice stimulated tumor-associated macrophages to attract neutrophils to the tumor, leading to tumor regression. These effects of the phage treatment were myeloid differentiation primary response 88 (MyD88)-dependent, suggesting activation of Toll-like receptors (TLRs) by the phages [37]. It was also shown that M13 phage DNA can trigger a type I IFN response in mice in vivo conferring protection against vaccinia virus infection [38]. In contrast, immunomodulatory and anti-inflammatory effects of phage preparations on immune cells have been observed in vitro [39] and in mice in vivo after systemic administration [40], but the mechanisms have not been worked out in detail [41]. Given the controversial results and lack of in depth studies, there is currently no clear evidence that resident enteric bacteriophages modify intestinal homeostasis and immune responses directly by interacting with eukaryotic cells in the intestine. It is rather more likely that the major effect of enteric phages is the modulation of the bacterial microbiome on the intestinal mucosal surface. This is also supported by the observation that the expansion of *Caudovirales* bacteriophages correlates with dysbiosis in IBD patients [9].

Studies in immunocompromised animals and patients show that mainly the eukaryotic viruses residing in the intestine are under constant surveillance by the immune system. Therefore, eukaryotic viruses should for now be considered the primary modulators of intestinal homeostasis and immune responses within the enteric virome.

It is not yet understood which cellular and molecular interactions are involved in the beneficial and/or detrimental effects of enteric viruses on intestinal homeostasis and stress resistance. To fully comprehend the role of the enteric virome in health and disease, it is crucial to obtain a comprehensive view of which host cell types and signaling cascades are employed in sensing and eliciting responses to enteric viruses. Another important question is which types of innate and adaptive immune responses against enteric viruses are generated and how these influence intestinal physiology.

## 2. Virus Sensing in the Intestine—Signaling and Subsequent Orchestration of Immune Responses

### 2.1. The Role of Pattern Recognition Receptors in the Intestine

PRRs include membrane bound TLRs and C-type lectin receptors (CLRs) and cytosolic nucleotide-binding oligomerization domain (NOD)-like receptors (NLRs), retinoic acid-inducible gene I (RIG-I)-Iike receptors (RLRs), and DNA sensors, such as absent in melanoma (AIM) 2-like receptors (ALRs) and cyclic GMP-AMP synthase (cGAS). These sensors, which recognize highly conserved microbial- or pathogen-associated molecular patterns (MAMPs or PAMPs) and trigger canonical innate immune response pathways, are important for the continuous immunosurveillance of tissues to detect the presence of microorganisms. Sensing of microbes by PRRs in the intestinal mucosa or other body surfaces is tightly controlled to avoid detrimental immune responses against harmless commensals and allows the generation of effective immune responses against invasive pathogens at the same time. Extending beyond this, innate and in some cases adaptive immune responses triggered by PRR activation shape the composition and function of the intestinal microorganisms to preserve a healthy microbiome. Thus, recognition of the components of the intestinal microbiome by PRRs is essential for maintaining homeostasis between the host and the microbiome and preventing intestinal pathology. This was shown in studies using knockout mice for diverse PRRs and central components of their signaling pathways, which demonstrate that constant low-level stimulation of these receptors by the microbiome is required to maintain intestinal barrier integrity and intestinal immune homeostasis [42,43]. As described by Thaiss et al. [44] feedback loops between the host and the microbiome exist at the layer of the IECs themselves. Sensing of microbes or microbial metabolites directly triggers a transcriptional response in the IECs and release of cytokines, which in turn induce the production of antimicrobial peptides and mucus by the IECs themselves. Particularly Paneth cells, specialized IECs sitting in the Lieberkühn crypts of the gut lining are an important source of antimicrobial peptides such as defensins, lysozyme C and phospholipases [45]. But also the MNPs in the lamina propria generate important regulatory signals in response to microbiome components via PRRs. Innate lymphoid cells (especially ILC3) in the lamina propria integrate signals derived from MNPs (such as IL-1β and IL-23) by producing IL-22 which feeds back to the IECs [46] or by producing granulocyte-macrophage colony-stimulating factor (GM-CSF) which feeds back to MNPs to produce regulatory factors [47]. Microbial cues are passed on further to adaptive immune cells in the gut-associated lymphoid tissue (GALT) and the draining lymph nodes (LNs) by migrating dendritic cells (DCs) leading to the production of IgA and regulatory T cells (Tregs), thus establishing homeostatic immunity to the microbiome [48].

### 2.2. Receptors and Pathways Involved in Virus Sensing in the Intestine 

Virus sensing by receptors of the innate immune system can be cell intrinsic when the ligands are produced in the infected cell (for example, replication of viral DNA or RNA) or cell extrinsic, when the virus itself or infected dead cells are internalized into phagosomes or endosomes [49]. Cell-intrinsic virus recognition in infected cells is mediated by cytosolic nucleic acid sensors, which are ubiquitously expressed in both immune and non-immune cell types, including RNA sensors of the RLR family and cytosolic DNA sensors, such as cGAS [49]. Moreover, cytosolic NLRs appear to be involved in cell-intrinsic virus sensing and modulate the other virus sensing pathways [50]. In contrast, cell-extrinsic virus recognition is mediated by transmembrane receptors, mainly TLRs [51]. CLRs, which can bind viral glycans, also mediate cell-extrinsic sensing of specific viruses, as shown, for example, for the Clec5a mediated immune response to dengue virus and *Influenzavirus A* [52,53,54].

Despite individual differences between the receptors and cell-type specific differences, virus sensing triggers several major signaling pathways that lead to the nuclear factor-kappa (NF-κ)B dependent induction of proinflammatory cytokines such as IL-1β, IL-6, TNF-α, IL-12 and to the interferon regulatory factor (IRF) 3 and/or IRF7-dependent transcription of type I IFN genes [55]. Triggering of RLRs and NLRs additionally leads to activation of the inflammasome, which activates caspase-1 to cleave pro-IL-1β and pro-IL-18 into active IL-1β and IL-18. The dominant type I and type III IFN response is however the major hallmark of the innate antiviral immunity and distinguishes it from innate immune responses to bacterial, fungal and parasitic infections, in which type I and III IFNs play only a minor role in most cases.

Type I IFNs are crucial in the control of virus infections. They comprise 16 members, 12 IFN-α subtypes, IFN-β, IFN-ε, IFN-κ and IFN-ω, which bind to and activate IFN-α/-β receptor (IFNAR) to induce the JAK (Janus kinase)/STAT (signal transducer and activator of transcription) signaling pathway [56]. Binding to IFNAR leads to activation of members of the STAT family via phosphorylation of Janus kinase 1 (JAK1) and non-receptor tyrosine kinase 2 (TYK2) and the subsequent induction of IFN-stimulated genes (ISGs), which control virus replication and spreading by targeting many steps in the virus life cycle [57]. Furthermore, type I IFNs confer resistance to uninfected neighboring cells, mediate killing of virus-infected cells by activating natural killer (NK) cells and cytotoxic T cells (CTLs) and promote adaptive responses by inducing cytokines and chemokines [58,59]. Early experiments also showed that many cell types could express both type I and type III IFNs in response to viral stimulation. The type III IFN cytokine family consists of IFN-λ1 (IL-29), IFN-λ2 (IL-28A) and IFN-λ3 (IL-28B) [60,61], which exhibit antiviral activity in epithelial cells that highly express the IFN-λ receptor [62]. Recent studies highlight a crucial role of IFN-λ against viral infections of the gastrointestinal tract including MNV infection [63,64,65]. In addition to their antiviral activity, type I and type III IFNs modulate and downregulate immune responses and thereby protect the intestinal epithelial barrier [66,67].

### 2.3. Toll-Like Receptors Mediate Cell-Extrinsic Virus Recognition

Toll-like Receptors are transmembrane PRRs that comprise an ectodomain containing leucine-rich repeats (LRRs) for PAMP recognition, a transmembrane domain and a cytosolic Toll-IL-1 receptor (TIR) domain [68]. The TLR family comprises TLR1-10 in humans and TLR1-9 and TLR11-13 in mice. TLR1/2/4/5/6 are localized on the cell surface whereas TLR3/7/8/9 and murine TLR11/12/13 are localized in endosomes [69]. The endosomal TLRs 3/7/8/9 recognize nucleic acids. TLR3 senses viral dsRNA, TLR7/8 sense viral ssRNA and TLR9 senses unmethylated CpG motifs in bacterial or viral dsDNA [70]. TLR2, TLR4 and TLR10 have also been described to be involved in the recognition of specific viruses [71,72,73]. Ligand binding to TLR7/8/9 (and TLR1/2/4/5/6) leads to the recruitment of the adapter protein MyD88, while activation of TLR3 (and TLR4) leads to the recruitment of TIR-domain-containing adaptor-inducing interferon-β (TRIF) [74,75,76]. TRIF associates with tumor necrosis factor receptor-associated factor (TRAF) 3, which promotes TANK-binding kinase 1 (TBK1) and IκB kinase ε (IKKε) mediated IRF3-phosphorylation and induction of IFN-β [77,78]. TRIF also associates with TRAF6 and receptor-interacting proteins 1 and 3 (RIP1 and RIP3) to activate NF-κB and mitogen-activated protein kinases (MAPKs) to induce proinflammatory cytokines [79]. In addition, MyD88 association with TRAF6 triggers the activation of NF-κB and MAPKs to induce proinflammatory cytokines. TLR7/8/9 activation recruits MyD88 and interleukin-1 receptor-associated kinases (IRAK)-1, -2, -4 to form a complex with TRAF6, TRAF3, IRAK1, IKKα, osteopontin and IRF7 leading to phosphorylation and subsequent activation of IRF7 which induces transcription of type I IFN genes and massive secretion of type I IFN by plasmacytoid DCs (pDCs) [80,81,82].

### 2.4. Role of Toll-Like Receptors in Intestinal Homeostasis and Inflammation

Due to the constant presence of microorganisms containing TLR ligands in the gastrointestinal tract, it was of great interest whether TLRs are functionally expressed at the intestinal epithelial interface and which roles they play there. Among the 13 TLRs discovered, TLR1-TLR9 have been identified as being expressed in human IECs at low levels, however expression is adjusted during intestinal inflammation; e.g., TLR4 is upregulated in the intestinal epithelium during active ulcerative colitis and Crohn’s disease, whereas TLR3 is downregulated in active Crohn’s disease [83,84]. Interestingly Paneth cells respond in vivo with degranulation to Lipid A, a TLR4 ligand, independently of TLR4, but only in the presence of TNF-α, indicating a mediating role for TNF-α. The TLRs 1–3 and 5–9 are expressed in Paneth cells [85]. Oral administration of polyI:C (TLR3 ligand) and CpG (TLR9 ligand) in mice leads to a fast Paneth cell degranulation, whereas Flagellin (TLR5 ligand) induces a slower response [86]. In addition to IECs and Paneth cells, many TLRs are expressed in goblet cells and enteroendocrine cells as well as MNPs in the intestinal mucosa [87].

Studies in laboratory mice indicate that MyD88-dependent signaling downstream of the TLRs and tonic activation of NF-κB in IECs are critical for maintaining intestinal barrier defense against commensals and response to injury (e.g., during acute DSS-induced colitis, [43]) while uncontrolled activation leads to increased inflammation. TLR activation in the intestinal epithelium is controlled at several levels: distinct apical versus basolateral distribution and signaling, downregulation of components of TLR signaling pathways (e.g., downregulation of IRAK1 by microRNA 146a [88]) and inhibition of TLR signaling pathways (e.g., by single immunoglobulin IL-1R-related molecule (SIGIRR) [89]). Stimulation of apical TLR9, for example, fails to activate NF-κB and mediates tolerance to subsequent TLR stimulation [90]. SIGIRR, one of the major negative regulators of TLR signaling, is highly expressed in IECs and SIGIRR deletion increases susceptibility to intestinal inflammation [89], demonstrating the importance of tight regulation of TLR signaling. Although it has been shown that tonic TLR signaling in response to the resident microbiota is crucial to promote wound healing, a fine balance of pro- and anti-inflammatory signals in the colonic epithelium is essential to maintain intestinal homeostasis. Therefore, SIGIRR plays a critical role in modulating intestinal inflammation and in preventing the development of chronic inflammation or colitis-associated cancer.

Rakoff-Nahoum and co-workers were the first to show that mice with global deficiency in MyD88 develop more severe colitis with colonic bleeding and increased mortality after chemical injury by oral application of DSS. This phenotype was not due to overgrowth of commensal bacteria or increased infiltration of leucocytes, but rather to impaired resistance to injury [43]. Deletion of TLR4 [43,91], TLR2 [43,92], TLR5 [93] and TLR9 [90] as well as simultaneous deletion of TLR3 and TLR7 [12] led to disturbed epithelial homeostasis and enhanced susceptibility to acute DSS-induced colitis, however with less severe pathology than in MyD88-deficient mice. These results indicate that constitutive activation of selected TLRs by the microbiome is required for epithelial homeostasis and response to injury [87]. Furthermore, TLR activation promotes IEC survival [92], limits IEC proliferation [43], maintains apical tight junctions [92] and induces the production of tissue protective factors such as cytokines, heat shock proteins, trefoil factor 3 [43] and epidermal growth factor receptor ligands [94].

IEC specific deletion of MyD88, TRAF6, NEMO (regulatory subunit of the IKK complex) and IKKα/β led to impaired epithelial barrier function and enhanced DSS-induced colitis [95,96,97,98] (reviewed in [42]). Results regarding the relevance of MyD88-dependent signaling in IECs versus different immune cell types for resistance of the intestinal epithelium to injury are controversial. Kirkland et al. found that B-cell specific deletion of MyD88 had the greatest effect as compared to deletion in myeloid cells or DCs, which had intermediate effects and IEC-specific deletion, which only had minor effects [99]. Malvin et al. demonstrated that expression of MyD88 by myeloid cells is sufficient to rescue MyD88 knockout mice from the severe pathology observed after exposure to DSS [100]. These findings indicate that microbial sensing by innate and adaptive immune cells below the epithelial cell layer also play important roles for homeostasis and stress resistance of the intestinal epithelium.

The contribution of virus stimulation of TLRs for the described protective effects of TLR signaling in the intestinal mucosa is still unclear. The increased susceptibility to DSS-induced colitis in mice lacking TLR3 and 7, which primarily sense viruses, may indicate that sensing of resident enteric viruses by these receptors protects the epithelium from colitis [12]. This was also corroborated in humans by association of a TLR3 single nucleotide polymorphism (SNP) with higher risk for IBD and association of combined TLR3 and TLR7 genetic variants with a more severe clinical course of IBD in a small cohort of Korean patients [12]. In that study it was also shown that the oral application of combined TLR3 and TLR7 agonists or inactivated rotavirus particles protected mice against DSS-induced colitis. The protective effects of TLR3/7 stimulation were associated with a reduced expression of NF-κB regulated inflammatory genes in the colon and reduced proinflammatory cytokine production by CD11b^+^ cells from the colon of DSS-treated mice after incubation with TLR3/7 agonists [12]. Lack of TLR3 and TLR7 during DSS colitis abrogated IFN-β production of colonic pDCs in response to TLR3/7 agonists indicating that type I IFN produced by pDCs may be important to counterregulate inflammation in the colon [12]. Similarly, administration of individual synthetic ligands for TLR3 [101], TLR7 [102] and TLR9 [103] had protective effects against colitis in mice, which were also associated with the induction of type I IFNs. However, application of TLR9 ligands showed controversial effects on intestinal inflammation in mouse models. Obermeier and colleagues showed that prophylactic treatment conferred protection against colitis whereas treatment during colitis development aggravated inflammation [104,105]. TLR9 was also shown to mediate the beneficial effects of probiotics on colitis development by sensing by the probiotics’ DNA and these effects were type I IFN dependent [106]. A recent placebo-controlled clinical trial with 131 patients which tested topical application of a synthetic TLR9 ligand (DIMS0150 applied to the inflamed mucosa at week 0 and 4) for treatment of active moderate-to-severe ulcerative colitis did not show a significant difference between treatment and placebo groups for the primary endpoint clinical remission at 12 weeks after starting treatment, but a significantly higher percentage of patients showed histological improvements and reached clinical remission with mucosal healing after four weeks [107]. It can be speculated that resident enteric DNA viruses, which activate TLR9 and induce type I IFNs could mediate similar protective effects in the healthy intestine as synthetic TLR9 agonists applied topically.

Constitutive IFN plays a role in maintaining immune homeostasis in the intestine [67,108], as IFNAR knockout mice are less susceptible to DSS-induced colitis [103]. IFNAR signaling in innate immune cells was required for protection against T cell transfer colitis and expression of anti-inflammatory mediators including IL-1R antagonist. The increased T cell-mediated colitis activity in IFNAR knockout recipient mice could be reversed by Anakinra treatment [109]. Type I IFNs have been shown to protect IECs from apoptosis [103] and promote intestinal organoid growth as well as production of antimicrobial peptide antimicrobial regenerating islet-derived protein 3 gamma (Reg3γ) [110]. In line with these results, Sun et al. had shown that persistently elevated systemic type I IFN increased epithelial cell turnover in several tissues including the small intestine and accelerated wound healing in the intestine, thus linking viral infection with increased epithelial proliferation and repair of tissue injury. This effect of systemic type I IFN was mediated by a myeloid-epithelial cell cross talk involving the induction of ApoL9a/b in myeloid cells, which triggers ERK signaling in epithelial cells [111]. However, type I IFNs also have anti-proliferative effects in the epithelium, which ensure proper differentiation of IECs, preventing hyperproliferation and tumor development [112,113].

### 2.5. Cytosolic Nucleic Acid Sensors Mediate Cell-Intrinsic Virus Recognition 

Cell-intrinsic viral recognition is mediated by intracellular (mostly cytosolic) receptors from different families: DNA sensors (cGAS/STING, AIM2 and interferon-γ inducible factor 16 (IFI16)), RNA sensors of the RLR family (retinoic acid-inducible gene I (RIG-I) and melanoma differentiation-associated gene 5 (MDA5)), as well as the NLRs.

### 2.6. Cytosolic DNA Sensors

The major cytosolic DNA sensor, cGAS [114], generates cGAMP which binds to the adapter protein stimulator of interferon genes (STING), thereby activating TBK1 to phosphorylate IRF3 and induce type I IFNs, plays a role in maintaining the integrity of the gut epithelial barrier after irradiation and hematopoietic stem cell transplantation [110]. Further other studies demonstrated a higher susceptibility of STING knockout mice to colitis induced by DSS or T cell transfer, which correlated with defects in production of mucus and IgA as well as changes in ILCs and impaired Tregs indicating the relevance of this pathway for maintaining intestinal barrier function and the prevention of inflammation [115]. Furthermore, STING-deficient mice are highly susceptible to colitis-associated cancer due to deregulated NF-κB and STAT3 signaling [116].

DNA derived from commensal bacteria, endogenous DNA or DNA from enteric viruses could also trigger the cGAS/STING pathway. The cytosolic DNA sensors AIM2 and IFI16 have been reported to play a role in the detection of DNA viruses, especially herpesviruses [117,118,119], and elevated levels of AIM2 and IFI16 are observed in the mucosa of patients with active IBD [120]. The AIM2 inflammasome, which can be triggered by cytosolic DNA derived from bacteria or viruses and by nuclear DNA damaged by irradiation, protects mice from colitis through IL-18-dependent induction of antimicrobial peptides [121,122] as well as Akt-dependent induction of tight junction proteins [123].

### 2.7. Cytosolic RNA Sensors—The RIG-I-like Receptor Family

The RLRs are a family of DExD/H box containing RNA helicases that are cytosolic sensors for RNA. There are three family members: RIG-I [124], MDA5 [125] and laboratory of genetics and physiology 2 (LGP2) [126]. The expression of RLRs is further induced by type I IFN during viral infection [127]. All RLR members share a similar structure with a central helicase domain containing an ATP-binding motif and a C-terminal domain (CTD) that mediates binding of the RNA ligand and a N-terminal tandem caspase recruitment domain (CARD), which is only present in RIG-I and MDA5, whereas it is lacking in LGP2 [124,128,129,130]. The N-terminal tandem CARD is crucial for homotypic interaction with the CARD-containing adapter protein IPS-1 [131], also termed MAVS (mitochondrial antiviral-signaling protein) [132], VISA (virus-induced signaling adapter) [133] or CARDIF (CARD adapter inducing IFN-β) [134], to initiate downstream signaling. LGP2 is signaling-inactive and serves as a regulator of RLR activation and signaling [135]. RIG-I is essential for the production of type I IFNs following recognition of short dsRNA or 5′-triphosphate RNA present in RNA viruses (and in bacterial RNA), whereas MDA5 detects viral RNA from picornaviruses and synthetic long dsRNA of more than 2 kb length [136,137,138,139]. Binding of the RNA ligand to MDA5 and RIG-I leads to engagement of MAVS, which is anchored to the outer mitochondrial membrane to induce antiviral signaling leading to the activation of IRF3 and the subsequent production of type I IFNs [132] and type III IFNs [140,141]. In the context of viral infections in the intestine, RIG-I and MDA5 have been shown to play an important role in the innate immune response against rotavirus, a dsRNA virus that can directly infect IECs and lead to severe diarrhea [142]. However, UV-inactivated rotavirus leads to higher induction of type I IFN compared to untreated rotavirus, indicating viral interference with the IFN induction pathway [143].

The production of type I IFNs and other cytokines by bone marrow derived DCs in response to MNV was dependent on MDA5. Replication of MNV CW3 strain, which causes acute infection, was controlled by MDA5 in the intestine and in lymphoid organs after peroral infection [144]. In mice treated with antibiotics, infection with the CR6 strain of MNV, which persistently infects small numbers of intestinal epithelial cells [21], restored intestinal resistance to chemical injury and bacterial infection by inducing type I IFNs [11]. However, it was not investigated in this study which cells produce type I IFN and which receptor is used to sense persistent MNV. It was shown that MNV can persist in a small number of IECs by evading IFN-λ-mediated antiviral immunity [21]

Several studies have shown that apart from sensing viral pathogens, RIG-I and MAVS, which mediates RIG-I and MDA5 signaling, play a role in maintaining gut homeostasis and preventing intestinal inflammation. Mice lacking RIG-I spontaneously developed a colitis-like phenotype with increased susceptibility to DSS-induced colitis [145] and are more susceptible to infection with *E. coli* compared to wildtype (WT) mice due to decreased phagocytosis of bacteria [146]. The spontaneous colitis phenotype of RIG-I-deficient mice was associated with reduced G protein α i2 subunit (Gαi2) expression, regression of Peyer’s patches and overactivation of T cells resembling the phenotype of Gαi2 knockout mice [145]. It has been observed that RIG-I is downregulated in the intestinal epithelium of patients with Crohn’s disease suggesting that innate antiviral defense mechanisms and protective effects of RIG-I signaling in the intestine could be impaired in Crohn’s disease [147]. RIG-I suppresses the development of colon tumors in mice by promoting IgA production and IL-6-STAT3-dependent expression of Reg3γ thereby regulating the microbiome [97,147,148]. Li et al. compared the phenotype of mice lacking MyD88, MAVS or both signaling adaptors in the acute DSS-induced colitis model. Interestingly, MAVS-deficient mice had increased colitis severity compared to WT mice, but the disease was more pronounced in MyD88-deficient mice and fulminant in MyD88/MAVS double knockout mice, indicating that these two PRR signaling pathways are not redundant, but cooperate to protect the intestine against injury and inflammation [149]. The protective effect seen in this model is mediated by MAVS signaling in non-hematopoietic cells. Mice deficient in MAVS or RIG-I were also shown to be more susceptible to intestinal damage induced by irradiation or chemotherapy and developed more severe GvHD after allogeneic HSCT [110]. Systemic administration of the RIG-I ligand 5′-triphosphate RNA or cGAS/STING activating DNA partially rescued mice from GvHD after allogeneic HSCT and this effect was reversed by IFNAR blockade. Studies using intestinal organoid culture demonstrated a direct effect of type I IFN on organoid growth and production of the antimicrobial peptide Reg3γ [110,149]. Both studies showed that feces RNA transfected into macrophages or epithelial cell lines induces IFN-β expression in a RIG-I and MAVS dependent manner [110,149]. This activity was greatly reduced when RNA was isolated from feces of mice treated with antibiotics or germ-free mice [149], indicating a major role for bacterial RNA sensing by RIG-I. It is still possible that also bacteriophages and eukaryotic RNA viruses residing in the intestine contribute to constitutive RLR activation for protection of the intestinal barrier. Actually, none of the described studies reported the MNV status of their mouse colony. The question if the enteric virome is protective in the context of HSCT and GvHD or potentially pathogenic as suggested by the expansion of specific enteric viruses in HSCT patients with GvHD [28] remains to be investigated.

### 2.8. Role of NOD-Like Receptors in Virus Sensing

NOD-like Receptors comprise a large receptor family characterized by the presence of a conserved NOD motif [150]. Depending on the structure of the N-terminal effector domain, NLRs can be divided into the following subfamilies: acidic transactivation domain-containing subgroup (CIITA), CARD-containing subfamily (NOD1, NOD2, NLRCs), pyrin domain (PYD)-containing subfamily (NLRPs) and baculoviral inhibitory repeat (BIR) domain containing subgroup (NAIPs) [151]. Activation of the NLRs occurs after sensing of a PAMP or danger associated molecular pattern (DAMP) by the C-terminal LRRs leading to the activation of diverse signaling pathways by the different NLRs. We will here focus on NLRs in the context of intestinal immunity and homeostasis and the potential link to the virome. Indeed, NLRs activate the inflammasome not only in response to bacterial ligands but also in response to viruses and are important modulators of other virus sensing pathways [152].

NOD1 and NOD2 were the first NLRs reported to function as intracellular microbial sensors [153]. NOD1 is widely expressed in many cell types and organs, while the expression of NOD2 is restricted mainly to DCs, macrophages, Paneth cells and IECs. NOD1 and NOD2 are known to recognize different peptidoglycan components of bacterial cell walls, which leads to the induction of NF-κB and MAPK signaling and a robust inflammatory response (summarized in [153]). Stimulation of NOD1 or NOD2 results in the secretion of proinflammatory cytokines and chemokines and production of antimicrobial peptides by IECs. Mice lacking NOD1 or NOD2 or both are highly susceptible to DSS-induced colitis and develop dysbiosis. Mutations in NOD2 have been linked to an increased risk of Crohn’s disease [154,155]. Apart from the essential role of the NODs in the regulation of the intestinal epithelial barrier function and the microbiome, there is evidence that they are also involved in the detection of viruses indicating a potential role for interaction with the enteric virome. It has been shown that NOD2 can also function as viral PRR by triggering activation of IRF3 and subsequent production of IFN-β via engagement of the RLR adapter protein MAVS in response to infection with respiratory syncytial virus (RSV) [156]. In addition, another NLR family member, NLRX1, has been identified to function as in vivo negative regulator of RLR and TLR signaling [157,158]. NLRX1 does not exhibit inflammasome function and is uniquely localized to the mitochondria [159,160]. NLRX1 attenuates TRAF6, MAVS/RIG-I, IRF3 and IκB kinase signaling in response to viral infection and TLR signaling [157,158,159]. The negative regulatory function of NLRX1 on RLR signaling does not rely on ligand competition, but instead on targeting the RLR downstream signaling adapter MAVS [159]. Lack of NLRX1 resulted in the production of higher amounts of IFN-β and IL-6 upon infection with viruses that trigger the RIG-I signaling pathway [157]. In this study, mice lacking NLRX1 had decreased viral titers in the lung after influenza infection due to an increase in IFN-β compared to WT animals but showed more severe lung pathology most likely because of exacerbated proinflammatory cytokine production. Allen et al. also observed that stimulation of macrophages lacking NLRX1 with the TLR4 agonist LPS resulted in elevated amounts of IFN-β and IL-6, suggesting that NLRX1 exerts a dual negative regulatory role in both RLR and TLR signaling. In contrast to the results described above, another group generated NLRX1-deficient mice, which did not display significant defects in RLR signaling [161]. Although it seems clear that NLRX1 functions as modulator of PRR signaling, rather than an actual receptor or sensor, further studies are needed to fully understand its function. However, NLRX1 has been shown to act as tumor suppressor in the colitis-associated cancer model in mice exerting its impact primarily through non-hematopoietic cells such as colon crypt cells [162], indicating a potential role for maintenance of gut homeostasis. Expression analyses of clinical human colon cancer samples revealed that NLRX1 expression is significantly lowered in colorectal cancer when compared to normal colon tissues [162], indicating that loss of NLRX1 may aggravate human colon cancer and further pointing towards a crucial role of NLRX1 for intestinal homeostasis.

In addition to the NOD proteins, NLRP6 has been shown to be crucial for maintaining intestinal homeostasis and a healthy intestinal microbiome [163]. NLRP6 is predominantly expressed in IECs [164]. NLRP6-deficient mice are more susceptible to intestinal inflammation and to chemically induced colitis due to the important role of NLRP6 for mucosal self-renewal and proliferation [165,166]. Furthermore, NLRP6 participates in the steady state regulation of the intestinal microbiome, partly through basal secretion of IL-18 [163]. NLRP6 associates with the inflammasome adapter apoptosis-associated speck-like protein containing CARD (ASC) and induces caspase-1 dependent processing of pro-IL-18 and pro-IL-1β into active IL-18 and IL-1β [167]. IL-18 controls the expression of antimicrobial peptides and the secretion of mucus by IECs [44]. Apart from its crucial role in maintaining the gut microbiome and regulating antibacterial immunity, NLRP6 was recently shown to play a role in the control of enteric virus infections [168]. Mice lacking NLRP6 failed to control replication of encephalomyocarditis virus (EMCV) or MNV in the gastrointestinal tract [168]. Interestingly, NLRP6 was activated by viral RNA which bound to the RNA helicase Dhx15 and interacted with the RLR signaling adapter MAVS to induce both type I and type III IFNs as well as ISGs. A recent study revealed that NLRP9b, which is specifically expressed in IECs, limits the replication of rotavirus [169]. NLRP9b recognizes short dsRNAs derived from rotavirus (but not long dsRNA such as from EMCV) indirectly via the RNA helicase Dhx9 and then forms the inflammasome, leading to release of active IL-18 as well as pyroptosis of the infected cell. Thus, rotavirus infected IECs are expelled prematurely from the epithelium to restrict replication and spreading of the virus [169]. A similar mechanism of expulsion of infected or neoplastic cells from the intestinal epithelium has been described for NLRC4, which senses flagellin and bacterial secretion systems [44,170]. Expulsion of infected cells could also be a relevant mechanism for controlling MNV or other viruses infecting IECs. The recent discoveries on NLRP6 and NLRP9b [168,169] show that, unexpectedly NLRPs via their interaction with DEAH-box (DHX) containing RNA helicases play an important role in the response to enteric RNA viruses.

**Figure 1:**
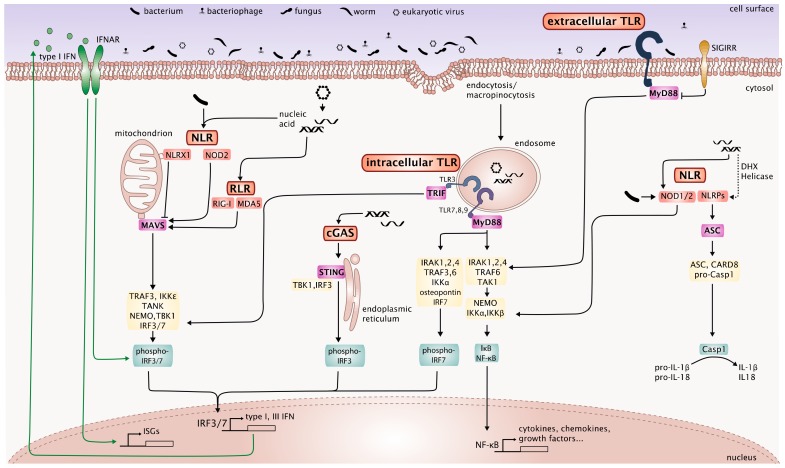
MAMP/PAMP sensing pathways in the intestine. Several PRRs are involved in sensing MAMPs/DAMPs in the intestine: TLRs, RLRs and NLRs. MAMPs/PAMPs are sensed either extracellularly by TLR1/2/4/5/6, or intracellularly by the endosomal TLRs 3/7/8/9 and cytosolic NLRs, RLRs or the cGAS/STING pathway. The receptors (red) signal via adapter proteins (magenta) and further downstream signal components (yellow) to the final players of the respective pathway (green), leading to gene expression or activation of effector molecules. Ligand binding to the extracellular TLRs and the endosomal TLRs 7,8,9 induces signaling via the adapter MyD88, leading to the recruitment and complex formation of IRAK-1/2/4 with TRAF6, TRAF3, IRAK1, IKKα, osteopontin and IRF7. Upon phosphorylation, IRF7 is activated and translocates into the nucleus to induce transcription of type I and III IFN genes. MyD88 signaling can be inhibited by the receptor SIGIRR. In addition, MyD88 leads to the formation of a complex comprising IRAK-1/2/4, TRAF6 and TAK1, which activates the IKK-complex (comprised of IKKα, IKKβ and NEMO), triggering degradation of IκB and allowing NF-κB to translocate into the nucleus to induce the transcription of proinflammatory genes. The cytosolic sensor cGAS signals via the adapter STING, which forms a complex with TBK1 and IRF3. TBK1 phosphorylates and thereby activates IRF3, leading to the transcription of type I and III IFN genes in the nucleus. RLRs, such as RIG-I and MDA5, employ MAVS as signaling adapter, which triggers the formation of a signaling complex containing TRAF3, IKKε, TANK, NEMO, TBK1 and IRF3/7. Subsequent phosphorylation of IRF3/7 leads to the transcription of type I and III IFN genes. The same complex is induced by TRIF signaling upon ligand binding to TLR3. The signaling adapter MAVS can be inhibited by the NLR family member NLRX1, which plays a modulating role. The other members of the NLR family activate different downstream signaling cascades. NOD2 can employ the RLR adapter MAVS, leading to type I and III IFN gene transcription or signal via the aforementioned IKK-complex, leading to NF-κB dependent production of proinflammatory cytokines. In contrast to the other nucleic acid sensors, NLRPs do not sense pathogen-derived nucleic acids directly, but employ DHX helicases (NRLP6: DHX15, NLRP9b: DHX9). NLRP signaling requires the adapter protein ASC. Activation of the NLRP pathway leads to the formation of a complex containing ASC, CARD8 and pro-Casp1, resulting in auto-activation of pro-Casp1 into the proteolytically active Casp1. In contrast to the other pathways mentioned, NLRP signaling does not lead to the activation of transcription factors, but to the maturation of the cytokines IL-1β and IL-18 by Casp1 mediated cleavage. Used abbreviations in alphabetical order: ASC—apoptosis-associated speck-like protein containing CARD; CARD—caspase activation and recruitment domain; cGAS—cyclic GMP-AMP synthase; DHX—DEAH-box; IKK—IκB kinase; IRAK—interleukin-1 receptor-associated kinase; IRF—interferon regulatory factor; IκB—nuclear factor of kappa light chain enhancer of activated B-cells inhibitor; MAMP/PAMP—microbial or pathogen-associated molecular pattern; MAVS—mitochondrial antiviral-signaling protein; MDA5—melanoma differentiation-associated gene 5; NEMO—NF-κB essential modulator; NF-κB—nuclear factor of kappa light chain enhancer of activated B-cells; NLR—Nod-like receptor; NLRP—pyrin domain (PYD)-containing subfamily of NLRs; NLRX1—NLR family member X1; NOD—nucleotide-binding oligomerization domain-containing protein; Casp1—Caspase 1; RIG-I—retinoic acid-inducible gene I; RLR—RIG-I like receptor; SIGIRR—single immunoglobulin IL-1R-related molecule; STING—stimulator of interferon genes; TAK1—transforming growth factor β-activated kinase 1; TANK—TRAF family member-associated NF-κB activator; TBK1—TANK-binding kinase 1; TLR—Toll like receptor; TRAF—tumor necrosis factor receptor-associated factor.

The complex crosstalk between the described PRR signaling pathways that are involved in innate immune responses to the microbiome, maintenance of the epithelial barrier function and homeostasis is depicted in Figure 1.

## 3. Intestinal Mononuclear Phagocytes Orchestrate Enteric Immune Responses

IECs themselves can directly respond to commensal bacteria and viruses and contribute to the production of type I and type III IFNs in the intestine in the steady state, and in response to viral infection as a first line of defense [67,171]. However, the maintenance of intestinal immune tolerance in the face of constant stimulation by commensal microbes as well as the development of effective immune responses against enteric pathogens including viruses also requires the specific function of MNPs in the lamina propria and in the GALT [172].

MNPs are present throughout the intestine and comprise different subsets, which form a complex network, whose functionality is crucial for intestinal homeostasis and protection against invading pathogens. MNPs not only sense environmental signals including microbes and initiate innate cytokine and chemokine responses, but they also interact with ILCs as well as T and B lymphocytes and thus shape the adaptive immune response in the intestine. Disturbance of the MNP network and their interplay with ILCs and the adaptive immune system can lead to intestinal inflammation or failure of the immune defense against enteric pathogens [173,174].

### 3.1. Intestinal Dendritic Cells

Dendritic cells are subdivided in two main groups, namely conventional and plasmacytoid DCs. Conventional DCs (cDCs) in general are especially capable of sampling and presenting endogenous and exogenous antigens to naïve T cells. Their ability to migrate from peripheral tissues to draining lymph nodes allows them to carry antigens to the T cell area of the lymph node, where antigen presentation takes place. Further, DCs shape the effector T cell differentiation by the secretion of cytokines. Plasmacytoid DCs (pDCs) have a unique capacity in rapidly producing large amounts of type I IFNs in response to viral infections [80], although they can also present antigens when they are specifically activated. In the following, the intestinal DC subtypes will be discussed in the light of their role in viral infections. The general development, phenotypical and functional features of intestinal DCs have been extensively reviewed elsewhere [175,176]. We will therefore give a short overview of the different subpopulations and focus on their potential function in the response to enteric viruses and their involvement in intestinal homeostasis and inflammation, which has been mainly investigated in mouse models, which lack specific DC subsets or allow selective ablation of DC subsets.

Conventional DCs are found throughout the intestinal lamina propria, as well as in isolated lymphoid follicles and Peyer’s patches. As they are located deep in the lamina propria without direct access to the lumen, several mechanisms come into play for sampling insoluble luminal antigens. Goblet cells, tissue resident macrophages, as well as specialized epithelial cells in the small intestine (villus M-cells) function as passage ways, which deliver antigen from the lumen to DCs in the lamina propria [177,178,179,180]. Moreover, Farache and colleagues observed *Salmonella* induced cDC migration inside the epithelial layer and direct sampling of luminal antigens [181]. After antigen-uptake, cDCs in the lamina propria migrate via the afferent lymphatics in a chemokine receptor (CCR) 7-dependent manner to the draining mLNs where they present the sampled antigens to T cells [182,183].

The importance of cDCs for resistance against intestinal inflammation was demonstrated in studies using CD11c-DTR mice, in which cDCs can be depleted by injection of diphteria toxin (DT) [184,185]. Furthermore, deletion of MHC class II expression specifically in cDCs results in chronic intestinal inflammation driven by commensal bacteria demonstrating that innate functions of DCs alone are not sufficient to maintain immune tolerance and to prevent inflammation in the intestine [186].

Intestinal cDCs are distinguished from macrophages by lack of CD64 and other monocyte/macrophage markers. To separate the two main cDC subsets in the intestine CD103, CD11b (in mice), XCR1 and SIRPα (in mice and humans) are commonly used as markers [187,188,189,190,191,192]. CD103^+^CD11b^−^ (murine) or CD103^+^ SIRPα^−^ (murine and human) cDC1 [192], which also express XCR1 as a specific marker [193], are mainly responsible for the cross-presentation of exogenous non-cytosolic antigens to CD8^+^ T cells. They correspond to the CD8α^+^ cDC1 in the spleen, which were identified as the major crosspresenting DCs [194] and also shown to be responsible for crosspriming of naïve CD8^+^ T cells for induction of tolerance in the steady state and induction of protective immunity after vaccination [195,196]. cDC1 are equipped with a restricted set of TLRs. TLR3 and TLR10 are highly expressed, TLR1-2, 6 and TLR8 at a low level and TLR7 is not expressed, though Fujimoto et al. found a small subset of cDC1 in the intestine, expressing TLR7 [197,198]. TLR9 is expressed in murine but not in human cDC subsets [199,200]. Moreover, the expression of two barely characterized TLRs—TLR12 and 13, which are expressed in mice but not in humans—is mainly restricted to the cDC1 subset [201]. TLR12 has been shown to play a role in sensing of parasites [202], TLR13 in sensing of bacterial 23S ribosomal RNA [203].

XCR1^+^ cDC1 have been shown to play an important role for intestinal T cell homeostasis because mice with constitutive ablation of XCR1^+^ cDCs have greatly diminished levels of intraepithelial lymphocytes (IEL) and lamina propria T cells, which goes along with an increased susceptibility to DSS-induced colitis [204]. Similarly, specific depletion of XCR1^+^ CD103^+^ CD11b^−^ cDC1 but not CD103^+^ CD11b^+^ cDC2 aggravated colitis in a low dose DSS model [205]. Mice with IRF8-deficiency in CD11c^+^ cells, which lack XCR1^+^ CD103^+^ CD11b^−^ cDC1, also had greatly reduced numbers of CD8αβ^+^ and CD4^+^ CD8αα^+^ T cells in the IEL fraction of the small intestine and lacked intestinal Th1 cells [206]. Thus, IELs appear to rely on signals from XCR1^+^ CD103^+^ CD11b^−^ cDC1 for their maintenance. This could be highly relevant for the antiviral defense in the intestine as activation of IELs was shown to protect IECs against viral infection by producing type I and type III IFNs, which induced the expression of ISGs in IECs [207]. cDC1 were also shown to be an important source of type III IFN (IFN-λ) in response to TLR3 stimulation in vivo and in vitro [208]. Interestingly, IRF8-dependent cDC1 in draining lymph nodes were found to serve as a platform for CD4^+^ T cell help for CD8^+^ T cell responses to viral infection [209,210]. It was also shown recently that CD103^+^ CD11b^−^ cDC1, which are absent in *Batf3*^−/−^ mice, were required for the generation of rotavirus-specific CD8^+^ T cells in neonates [211]. Thus, IRF8-dependent cDC1 appear to play a major role for antiviral defense in the intestine and could also be involved in mediating tolerance to enteric viruses residing in the intestine as commensals.

Within the CD11b^+^ or SIRPα^+^ cDC2 in the intestine, the CD103^+^CD11b^+^ cells, which are the more frequent population in the small intestine [212], were shown to be critical for the induction of Th17 cells and ILC3 in mLNs and for the defense against fungal and bacterial infections [213,214,215]. However, also the CD103^−^CD11b^+^ cDC subpopulation, which is the more abundant population in the colon [212], was shown to be capable of inducing Th17 cells and ILC3 and therefore could be relevant for maintaining Th17 and ILC3 responses in the colon, with ILC3 important for supporting intestinal epithelial barrier function [216,217]. Both CD103^+^ CD11b^+^ and CD103^−^ CD11b^+^ cDC are also important for inducing Th1 and Th2 responses in the intestine in the context of infection depending on the pathogen they encounter. The specific role of these intestinal cDC2 subpopulations in the response to enteric viruses has not been investigated. However, it was shown that migratory IRF4-dependent cDC2 are required for T follicular helper cell induction and antibody production for protection against influenza A virus infection in the lung [218]. cDC2 express all TLRs (with the exception of TLR9 in human cDC2), which enables them to sense a wide range of PAMPs/MAMPs, both extra- and intracellularly [197]. Concerning virus sensing it was shown that cDC2 not only express intracellular TLRs to sense viruses, but contrary to the cDC1 counterpart also cytosolic sensors like the NLR NOD1 and the RNA sensors RIG-I and MDA5 are highly expressed in cDC2 [201].

Plasmacytoid DCs are sparsely present in the intestine and appear to be mainly located in the small intestine where they home in a CCR9-dependent manner and contribute to intestinal immune tolerance, whereas they are almost absent in the colon in the steady state [219,220]. However, they are found in the inflamed colonic mucosa of IBD patients [221]. In macaques they were shown to migrate to the jejunum, colon and gut draining lymph nodes in case of a pathogenic SIV infection, [222,223,224]. There, they may contribute to chronic immune activation in the intestine [225], which is supported by observations in HIV-infected human patients [226].

In contrast to cDCs, pDCs are specialized in the expression of the endosomal TLRs with strong expression of TLR 7 and 9 [200,227]. Moreover, the activation induced pDC subsets show differential expression of TLR6, which is known to form a heterodimer with TLR2 for sensing of lactic acid bacteria with beneficial effects on epithelial cells [228,229]. Similar to cDC1, pDCs express the cytosolic sensors RIG-I and MDA5 [230,231] and the endosomal TLR12 [232]. pDC activation via TLR7 and 9 leads to secretion of type I and type III IFNs, as described above. Besides this main function, pDCs were found to directly activate T- and B cells in rotavirus infections [233,234].

Arimura and co-workers propose a role of pDCs in colitis development, as pDC-ablated mice showed ameliorated symptoms upon colitis induction with DSS, decreased production of inflammatory cytokines and a lower leukocyte influx into the colonic lamina propria. The effect was independent of type I IFN, however, the exact mechanisms underlying this phenotype remain unclear [235]. In response to oral administration of the TLR7/8 ligand R848 pDCs appear to induce migration of cDCs from the small intestine via afferent lymphatics to the mLNs [219,236], while pDCs themselves do not migrate to the mLNs [237]. Like other antigen presenting cells, pDCs can take up antigens by phagocytosis, receptor-mediated endocytosis and to a low extent by macropinocytosis [238,239,240]. The open questions which mechanisms are employed for the transfer of luminal antigens to pDCs sitting in the lamina propria, in Peyer’s patches or in mLNs as well as whether pDCs participate at all in presenting luminal antigens to T cells remain to be investigated.

The type I IFN producing capacity of Peyer’s patch pDCs in response to TLR9 stimulation was found to be reduced compared to splenic pDCs and exposure of splenic pDCs to TGF-β, IL-10 or PGE_2_ reduced their type I IFN response suggesting that type I IFN production by pDCs is downregulated in the intestinal microenvironment [241,242]. But nevertheless, pDCs were shown to produce type I IFNs also in response to enteric viruses, such as rotavirus [243]. pDCs also play an important role for IgA production by B cells in the GALT due to their expression of B cell activating factor (BAFF) and the proliferation inducing ligand (APRIL) which required type I IFN signaling [244]. However, a recent study which employed transgenic mouse models for both short-term and long-term depletion of pDCs showed that pDCs were not essential for intestinal IgA responses in the steady state [245], demonstrating redundancy with cDC subsets regarding intestinal B cell activation in the absence of infection [246,247]. In contrast, pDCs and pDC-derived type I IFN were found to be necessary and sufficient for B cell activation and specific IgA production in response to rotavirus infection as demonstrated by specific pDC depletion in vivo [248].

Moreover, pDCs were shown to have immunosuppressive functions and thereby contribute to oral tolerance and may alleviate colitis [249]. For example, it was found that pDCs detect polysaccharide A from *Bacteriodes fragilis* via TLR2 and induce Tregs, which produce IL-10 leading to protection against trinitrobenzenesulfonic acid (TNBS)-induced colitis [250]. In a recent publication, it was shown that upon activation human pDCs diversify into three subpopulations, of which one assumes potent antigen presenting function similar to cDCs, and one produces type I IFNs [228]. A specific type I IFN producing subset within pDCs has also been discovered using IFN-β reporter mice [251]. These studies suggest a further division of labor between functionally distinct subsets of pDCs, the significance of which remains to be investigated in the context of the local tissue environment and during infection.

The differential equipment with PRRs between the DC subsets highlights their division of labor in sensing and responding to pathogens such as viruses. Whereas cDC2 cells are infected by e.g., HIV and allow viral gene expression and replication, cDC1 as well as pDCs remain resistant against infection with many enveloped viruses by a mechanism, which prevents release of viruses from the endosomal compartment [252]. The infected cDC2 senses the replicating virus via its cytosolic nucleic acid receptors and secretes proinflammatory cytokines and type I IFNs, but its innate responses and capacity to present viral antigens are limited by viral interference with innate response pathways and by the cytopathic effect of the virus. However, cDC1, which are not productively infected, cross-present viral antigens derived from infected cDC2 or other bystander cells to T cells in order to evoke a virus specific T cell response [252]. Virus sensing by cDC1 via endosomal TLRs further promotes efficient cross-priming of CD8^+^ T cells [253]. During viral infection, activated pDCs cooperate with cDC1 in the lymph node to optimize their maturation and cross-presentation ability [254]. Furthermore, virus sensing by pDCs induces B cell activation and IgA secretion [248,252]. Thus, different DC subpopulation and virus sensing pathways cooperate in the induction of innate and adaptive antiviral immune responses.

### 3.2. Intestinal MNPs of the Monocyte-Macrophage Lineage

Unlike in other tissues, adult intestinal macrophages (Mϕ) are mostly of hematopoietic origin and derived from infiltrating inflammatory monocytes [255]. When they enter the intestinal tissue, a 5 to 7 day long differentiation process to resident macrophages begins, in which first MHC class II expression is gained and then Ly6C expression is lost [256]. Resident macrophages have important regulatory functions in the intestine by the secretion of cytokines, such as IL-10, controlling inflammatory monocyte influx or production of IL-1β, which is critical for the survival of Th17 cells [257,258]. In inflammatory situations, this differentiation process fails and monocytes are constantly recruited to the site of inflammation, so that early differentiation stages become overrepresented [259]. Monocytes and immature Mϕ are potent producers of proinflammatory cytokines and inflammatory mediators [260]. The importance of tight regulation of the proinflammatory capacity of these cells in the intestine is shown in mouse models with myeloid cell specific deficiency of IL-10 receptor (IL-10R) or STAT3, which is required for IL-10R signaling [261,262]. In these mouse models as well as in patients with very early onset of IBD due to mutations in IL-10 or IL-10R [263], uncontrolled production of proinflammatory mediators, especially IL-1β [264] leads to severe intestinal inflammation. Intestinal Mϕ must be distinguished from CD11b^+^ cDCs by the expression of the F_c_ receptor CD64, as they can express all other known surface markers of the CD11b^+^ CD103^−^SIRPα^+^ cDC. This similarity is also found on a functional level, as intestinal Mϕ are also able to induce Th17 and ILC3 [265]. Additionally, they are crucial for the maintenance of intestinal homeostasis either directly by promoting tissue repair [266] or by promoting immune tolerance, for example, by transferring luminal antigens to cDCs [179]. The intestinal macrophages achieve this by projecting protrusions between epithelial cells into the lumen. Thereby they can pick up luminal antigens and pass them on to tolerogenic CD103^+^ cDC1, which induce Tregs [179]. In addition, they can clear bacteria before they can enter the lamina propria [267].

Mϕ are equipped with a range of TLRs, both extra- (TLR1, 2, 4, 6) and intracellularly (TLR3, 7, 9) [268,269,270,271,272], as well as cytosolic nucleic acid recognition receptors, making them powerful detectors of PAMPs. Intestinal Mϕ were shown to be readily infected by HIV and serving as reservoir for the virus in its latency phase [273]. Rotavirus infection of macrophages led to CXCL2 secretion and subsequent neutrophil recruitment, indicating a role in immune cell recruitment to the infection sites [274]. Though Mϕ are specialized in phagocytosis and are poor antigen presenting cells compared to cDCs, they have been shown to play a crucial role in infection with Enterovirus 71 by activating invariant natural killer T (iNKT) cells [272,275]. Most likely their inferior presentation capacity is due to higher protease levels in their lysosomal pathway resulting in stronger antigen degradation in Mϕ compared to DCs [276]. In addition to recruiting immune cells by chemokine secretion, intestinal Mϕ play a role in viral infections via their positive effect on epithelial cells, mentioned before. They induce proliferation and support survival in epithelial cells by mediators like Wnt signaling ligands, as well as tissue remodeling by secretion of metalloproteinases and TNFα [266,267,277,278].

**Figure 2:**
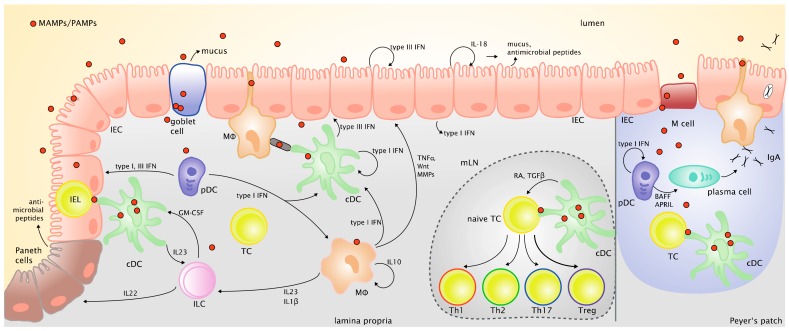
Antigen uptake routes and multicellular responses in the intestine. MAMPs/PAMPs are taken up from the intestinal lumen into the lamina propria via goblet cells or macrophages, which forward the antigens to cDCs, which then present the antigens to IELs or naïve TC in the mLN. There, they induce TC differentiation by expression of costimulatory molecules and cytokine secretion. Mϕ do not migrate to the mLN but secrete cytokines and factors, supporting tissue remodeling and repair. Autocrine IL-10 signaling keeps them in a hyporesponsive state, preventing excessive proinflammatory cytokine release. Type I IFN secreted by pDCs, cDCs and IECs acts on Mϕ, influencing their activation status, and on IEC and cDC themselves, inducing ISG expression (IECs) and cytokine secretion (cDCs). Autocrine type III IFN and IL-18 signaling is beneficial for IECs, as well as IL-22, secreted by ILCs, which in turn are influenced by cDC or macrophage derived IL-23 and IL-1β. In addition to IL-22, ILCs secrete GM-CSF that influences cDC phenotype and survival. In the Peyer’s patches of the small intestine, M cells and Mϕ provide antigens, which are presented to TC by cDCs. In the FAE of the Peyer’s patches pDC-derived BAFF and APRIL promotes IgA production by plasma cells. Used abbreviations in alphabetical order: APRIL—proliferation inducing ligand; BAFF—B cell activating factor; cDC—conventional dendritic cells; FAE—follicle-associated epithelium; GM-CSF—granulocyte-macrophage colony-stimulating factor; IEC—intestinal epithelial cell; IELs—intraepithelial lymphocytes; IL—interleukin; ILCs—innate lymphoid cells; ISGs—IFN-stimulated genes; MAMP/PAMP—microbial or pathogen-associated molecular pattern; mLN—mesenteric lymph node; Mϕ—macrophage; pDC—plasmacytoid dendritic cell; TC—T cell.

Figure 2 provides an overview of the various cell types and mediators that are critical in shaping epithelial barrier function and immune responses against intestinal antigens.

## 4. Context Dependent Sensing of the Enteric Virome—Summary and Future Perspectives

Studies performed in the last years have shown that the viral component of the microbiome, the enteric virome, may have an important role in maintaining intestinal homeostasis, but can also become pathogenic in situations of a breakdown of local defense mechanisms and control by the immune system. We have summarized here the current knowledge on receptors, signaling pathways and cellular players involved in sensing viruses in the intestine and have discussed the few studies which provide evidence that some of these pathways also play an important role for mediating the responses to viruses residing in the intestine of the healthy organisms, thereby shaping intestinal physiology. It becomes clear from the existing data that virus sensing pathways form a complex network and cross-regulate each other to adjust responses. The differences between specific epithelial and immune cell populations in the expression and activation of these virus sensing pathways add another layer of complexity. Moreover, an important aspect is also the observation that constitutive activation of PRRs induces responses in the intestine, which in turn shape the composition of the microbiome including bacteria, viruses and fungi. Thus, the beneficial but also the detrimental effects of the enteric virome are dependent on the context of the local intestinal milieu and the complex interaction with other microorganisms residing in the intestine.

Several issues remain to be resolved regarding the impact of the enteric virome on intestinal homeostasis and immune responses. First of all, it is necessary to clearly separate effects of the enteric virome and the bacterial microbiome to investigate if the enteric virome has a non-redundant role in regulating homeostasis in the intestine. Furthermore, the specific contribution of different enteric virus strains, which reside in the intestine remains to be investigated. It will be challenging to establish in vitro culture systems to propagate these viruses, which is a prerequisite to perform controlled infection experiments with individual virus strains in vitro and in vivo. The availability of such isolated virus strains would allow to investigate the cellular tropism of enteric viruses and the virus-host interactions that enable them to reside in the intestine as commensals for longer time periods. MNV infection is a great model to study infection and persistence of an enteric virus in the murine intestine and its effects on intestinal physiology as well as its modulation of the intestinal immune system. But, clearly, further models need to be studied to discover common effects of resident enteric viruses and specific effects of individual viruses residing in the intestine. As shown by the discovery of MNV, the use of immunodeficient mice may be useful to discover novel eukaryotic enteric viruses, which could serve as suitable models.

To investigate effects of the enteric virome, we can try to copy reductionist approaches, which have been performed to study effects of the bacterial microbiome, such as using germ-free or antibiotic treated mice. An approach, which mimics the depletion of intestinal bacteria by antibiotic treatment would be oral and/or systemic application of antiviral drugs to investigate the role of enteric viruses. This approach has been tested and was found to increase colitis susceptibility in mice, however only a slight reduction in detectable viral sequences was observed, showing that enteric viruses have differential sensitivity to this treatment and broader acting antiviral agents need to be used. Additionally, microbiome changes were induced by a treatment making it difficult to separate effects of enteric bacteria and viruses [12]. Bacteriophages in the intestinal lumen influence the intestinal microbiota, but are probably not targeted by these drugs. Germ-free mice can be used to study the consequences of infection with resident enteric viruses on intestinal physiology and immunity, as was shown for infection with a persistent MNV strain [11]. However, it would be important to verify first, that germ-free mice are also “virus-free” by sequencing the luminal contents, before inoculating them with individual or combined enteric virus strains, which have been found to reside in the intestine of mice.

The use of genetically modified mice especially cell type-specific and conditional knockout for specific virus sensors and signaling adaptors is a valuable approach to understand the complex host response to the enteric virome and the contribution of specific pathways to the beneficial and detrimental effects of enteric viruses. For the correct interpretation of these future studies it will be critical to perform experiments with co-housed or separately housed littermate controls to separate effects of the host genotype on the microbiome from the actual effects of the host genotype. Furthermore, it would be necessary at this point to always disclose the MNV status of the mouse colony when reporting experiments, in which effects of the enteric virome are investigated, especially when using mice lacking molecules important for innate or adaptive immune responses.

The species-specificity of eukaryotic enteric viruses is suspected to be an obstacle for transferring insights from the mouse model to the human situation. It is challenging to stably infect mice with commensal enteric viruses found in humans. To investigate effects of the human enteric virome in vivo, a switch to another animal model might be warranted. For example, the germ-free or gnotobiotic pig-model can be colonized more easily with the human microbiota and whose intestinal physiology and immune system is very similar to that of humans. The enteric virome in pigs appears to be similar to that of humans [279] and gnotobiotic pigs can be infected with human noroviruses and rotaviruses, for example [280].

Studies performed so far clearly show that the effects of the enteric virome are dependent on the context of host genetic variation and composition of the whole microbiome as bacterial, viral and fungal components of the microbiome will influence each other and all components of the microbiome are under the tight control of local defense mechanisms as well as immune responses. Which immune responses have a major influence on the abundance and composition of the enteric virome? Regarding local defense mechanisms, type I and especially type III IFNs appear to be important for controlling the replication of enteric viruses. Viral interference with induction and signaling of these antiviral IFNs in intestinal host cells is important for allowing persistence and shedding of the enteric viruses and for the establishment of a symbiotic relationship with the host. It has not been investigated which virus sensing receptors and downstream signaling pathways are crucial for controlling the enteric virome. Studies using pathogenic enteric viruses (i.e., rotavirus or EMCV) demonstrate that several pathways are activated upon viral infection of intestinal host cells; these pathways influence each other to adjust the response to the local requirements of the intestine. Evidence from human and animal studies indicate that in addition to innate immunity also adaptive immune responses exert control over the enteric virome to preserve the symbiosis with the host in the intestine. Future investigations will reveal which resident enteric viruses are capable of inducing these adaptive immune responses, which antigen-presenting cells present the viral antigens, and what is the outcome regarding T and B cell differentiation. A thorough understanding of enteric virome sensing and its subsequent effects on homeostasis and immune responses in the intestine is critical for harnessing the enteric viromes protective capacity or for designing drugs that mimic its beneficial effects on both intestinal barrier function and immune tolerance, for example, in the context of infection and inflammation.
